# Blockchain-Secured Recommender System for Special Need Patients Using Deep Learning

**DOI:** 10.3389/fpubh.2021.737269

**Published:** 2021-09-20

**Authors:** Eric Appiah Mantey, Conghua Zhou, Joseph Henry Anajemba, Izuchukwu M. Okpalaoguchi, Onyeachonam Dominic-Mario Chiadika

**Affiliations:** ^1^School of Computer Science & Comm. Engineering, Jiangsu University, Zhenjiang, China; ^2^Department of Communication Engineering, Hohai University, Changzhou, China; ^3^Department of Computer Science, Nnamdi Azikiwe University, Awka, Nigeria; ^4^Electronic and Computer Engineering, Brunel University, London, United Kingdom

**Keywords:** blockchain privacy system, deep learning, machine learning, IOMT, artificial intelligence

## Abstract

Recommender systems offer several advantages to hospital data management units and patients with special needs. These systems are more dependent on the extreme subtle hospital-patient data. Thus, disregarding the confidentiality of patients with special needs is not an option. In recent times, several proposed techniques failed to cryptographically guarantee the data privacy of the patients with special needs in the diet recommender systems (RSs) deployment. In order to tackle this pitfall, this paper incorporates a blockchain privacy system (BPS) into deep learning for a diet recommendation system for patients with special needs. Our proposed technique allows patients to get notifications about recommended treatments and medications based on their personalized data without revealing their confidential information. Additionally, the paper implemented machine and deep learning algorithms such as RNN, Logistic Regression, MLP, etc., on an Internet of Medical Things (IoMT) dataset acquired *via* the internet and hospitals that comprises the data of 50 patients with 13 features of various diseases and 1,000 products. The product section has a set of eight features. The IoMT data features were analyzed with BPS and further encoded prior to the application of deep and machine learning-based frameworks. The performance of the different machine and deep learning methods were carried out and the results verify that the long short-term memory (LSTM) technique is more effective than other schemes regarding prediction accuracy, precision, F1-measures, and recall in a secured blockchain privacy system. Results showed that 97.74% accuracy utilizing the LSTM deep learning model was attained. The precision of 98%, recall, and F1-measure of 99% each for the allowed class was also attained. For the disallowed class, the scores were 89, 73, and 80% for precision, recall, and F1-measure, respectively. The performance of our proposed BPS is subdivided into two categories: the secured communication channel of the recommendation system and an enhanced deep learning approach using health base medical dataset that spontaneously identifies what food a patient with special needs should have based on their disease and certain features including gender, weight, age, etc. The proposed system is outstanding as none of the earlier revised works of literature described a recommender system of this kind.

## Introduction

The idea of a secured recommendation system has been proposed in recent times due to the nature of diseases and dietary effects suffered by patients with unique conditions that inhibit their ability to receive routine diet recommendation care. This system which is incorporated with a blockchain privacy mechanism is advantageous to both the hospital data management unit and the patients with special needs in terms of privacy violation protection, scandals, and longevity of the patients. In (1), the authors gave a comprehensive explanation, including their history and basic concepts of networks and how they could be applied in the field of pharmaceutical sciences as an option instead of just traditional methodology. The authors of (2) discussed the various ways Artificial Neural Networks (ANNs) can be applied to resolve challenges in the pharmaceutical field. Similarly, in (3), the authors discussed how ANNs can be used in diverse ways in medicine such as in the delivery of drugs, classification of cancer, research in pharmaceutics and others. The authors of (4) proposed an algorithm to solve the issue of classification, analysis, and summarization of document analytics. Experimental results showed the efficiency of the algorithm regarding its precision and implementation time. In (5), the authors proposed a solution based on deep learning for medical datasets which identifies what kind of food a patient should be fed based on factors like the nature of diseases, gender, age, and weight, among others. Their framework made use of machine learning and deep learning algorithms. Experimental results showed its efficiency and accuracy.

The motivation of this study is to enhance, secure, and analyze the performance of an expansive nutritional theory into the Internet of Medical Things (IoMT) using a blockchain privacy system and deep learning algorithms.

The contributions of this study are stated below:

- To propose and incorporate a reliable blockchain privacy system (BPS) for a secured diet recommendation system for patients with special needs.
- To investigate and perform analysis of machine learning, deep learning system previously used on IoMT such as Naive Bayes and logistic regression, recurrent neural network (RNN), gated recurrent unit (GRU), and long short-term memory (LSTM).
- To design an all-inclusive diet recommendation model which can be applied for patient special needs products and disease specifications.
- To analyze the behavior of our Enhanced Artificial Intelligence (AI) and deep learning mechanisms and how it is administered.
- The result showcased the Enhanced Machine Learning and deep learning for patients with special needs and for treating varied patient diseases with varied recommender evidence that is secured using a blockchain privacy system.

### Structure

The remaining part of the study laid out on this paper is organized as follows. Several literature are revised and detailed in the related works section. Introduced in section System Model are the methods and materials of the system, including its execution utilizing AI. Summarized in section Experiments and Results are the findings of the conducted experiment in this study. Finally, the conclusions of the study are discussed in section Conclusion and Future Work.

## Related Works

Blockchain is one of the most recent subjects currently under-studied and has been recently incorporated into several societal, industrial and academic scenarios (6). One of the areas that have extensively incorporated the BPS is the hospital and patient recommender systems. The authors in (7) proposed research for customizing a service for the recommendation of diet for its customers which prevents and manages heart diseases in the area of healthcare. It provides this service by putting into consideration factors like family history of illnesses, preferences of food, vital signs, etc., of the customers with the issue of heart diseases. This service can aid customers to change their living habits to healthier ones. The study conducted by the authors of (8) proposed a framework for nourishment recommendation where input is retrieved from children and analyzed, which results in output that provides for a better and healthier diet plan. Its goal is to provide kids from the age of eight to their early teens with a healthier eating lifestyle which varies according to their age, gender, and other related factors. The study of the authors in (9) suggested a platform that smartly plans the meals of users according to their health conditions. This platform makes use of machine learning algorithms to function. The authors of (10) studied the impact of various factors which influence the adoption and use of a user of online health facilities, specifically in Ukraine and China. The authors in (11) proposed a framework that recommends healthy diet plans to patients that have hypertension. It utilizes machine learning algorithms and considers factors such as allergies, food preferences, age, and blood pressure, among others. The study conducted by the authors of (12) discussed how blockchain technology in conjunction with the Internet of Things (IoT) could be used to monitor the supply chain efficiently. The \ study of the authors in (13) proposed a system that recommends healthier diets specifically for American Indians with diabetes, by studying the profile of the user with clinical regulations and guidelines. The system makes customized recommendations based on various factors for the AI patient. The authors of (14) used a neural network deep learning method to explain how big data analytics could be utilized in the execution of an efficient system for health recommendation, and demonstrates how the industry of healthcare could evolve from a customary system to a more customized one in the online health environment.

The authors in (15) examined the ways for calculating and measuring the similarities of users in health websites. The recommendation of similar users to customers aids in the support search, whether emotional or not in a more efficient way. The study conducted by the authors of (16) presented an overview of the literature that examines how machine learning algorithms are used in recommender systems and also pinpoints opportunities for research in the software engineering field. It concluded that the algorithms are used because they are not complex. The study of the authors in (17) proposed a method for recognizing parameters that influence processes and suggests a framework for machines by executing a device that can collect a large amount of data and integrate them into the Cloud for more studies. The authors of (18) identified various ways big data analytics could be utilized and gave its benefits regarding IT framework in strategic areas. They also suggested tactics for organizations concerned with healthcare to adopt big data analytics. The authors in (19) proposed a recommendation of threads to health communities' online users by making use of diverse healthcare information network mining. The study conducted by the authors of (20) proposed a scheme to improve the efficacy of recommender systems and preserve privacy, as long as the data collected are examined efficiently. The study of the author in (21) provided an exhaustive review of all research regarding and related to recommender systems that are based on deep learning technology. The authors of (22) proposed a model that is based on fog deep learning that acquires data from people and forecasts their fitness statistics and abnormalities making use of a model that is based on a neural network that is capable of handling diverse and large amounts of data.

The authors in (23) proposed a recommender system that delivers accommodative nutrition information to extend and improve the lives of people with diet-related illnesses, as well as healthy people. The system recommends nutrition according to the health profile of the user. The study conducted by the authors of (24) proposed an architecture that collects and collates data from different primary indicators of performance in healthcare communities, as well as predicts possible values of these principal performance indicators. The study of the authors in (25) proposed a system that utilizes customized features concerning nutrition that aids users in the change of their attitude toward healthier food choices and diets. The authors of (26) conducted a review to determine if dieted and nutritional products are issues often left out in shortages research. The authors in (27) proposed a framework for the recommendation of everyday meals while also managing preference-inclusive and nutritional information. The study conducted by the authors of (28) proposed a recommender system making use of analysis of food clustering for patients with diabetes. The study of the authors in (29) reviewed numerous existing works of literature and discovered lacunas in the existing systems of diet management. The authors of (30) in their study concentrated on developing a recommender system that integrates Artificial Intelligence methods and creates a base of knowledge following the appropriate and lawful guidelines and regulations concerning diabetes. The recommended menu is according to the preferences and conditions of the patients.

The authors in (31) presented a recommender system that is based on the pathology report of the user. This system utilizes the ant colony algorithm to create a menu and suggests appropriate food in line with the values of the pathology report of the user. The study conducted by the authors of (32–34) discussed error sources regarding nutrition, as well as possible solutions to tackle those challenges for future systems. They also discussed the incorporation of nutritional propositions into information systems. The study of the authors in (35) proposed a management system to aid in the preparation of a proper and healthy diet for children. The authors of (36) studied the shifts in obesity and its various kinds from the richer to the less fortunate across an era of economic growth in a developing country (Malaysia). They concluded that as the country develops, the risk of obesity among the less fortunate should be expected. The authors in (37) proposed a customized recommender system fashioned to meet the nutritional restrictions, preferences, and expectations of the user. The conducted by the authors of (38) recommended a diet plan to patients with jaundice using a hierarchy process that considers vital nutrients to determine the best choice for meals for all times of the day. The study of the authors in (39) discussed the uses and benefits of deep learning methods and ANNs in the advancement of the medical field in areas such as electroencephalography and the collation of physiological data. On the other hand, the authors in (40) presented a review of recommendation methods for people and groups to eat healthier foods. They also studied the existing recommender systems and challenges regarding research of recommendation technology. The study of the authors in (41) presented a Privacy-Preserving and Secure Framework (PPSF) for IoT-driven smart cities. The proposed PPSF is subject to two key schemes: a two-level privacy mechanism and an intrusion detection mechanism. Finally, the potentials of blockchain-enabled edge-of-Things (BEoT) by Prabadevi et al. in (42) was designed to provide security and services which comprises trust management, attack detection, data privacy preservation and access authentication.

## System Model

The methodology used in this study is explained in the following discussions. The objective of this study is for the recommendation of diet to various patients with special needs with a BPS utilizing machine and deep learning classifiers for the health-based medical dataset which will spontaneously identify what kind of food a patient with special needs should have, based on their disease and other factors such as weight, gender, and age, among others. For this reason, we have incorporated a BPS utilizing various deep and machine learning classifiers. A random forest classifier was utilized to know what feature has a greater effect on the dataset.

### Dataset

The dataset utilized in this study comprises about a thousand products and 50 patients collated using IoT and cloud methods. The products experimented on various disease patients. The dataset possesses over 13 features and has stored in it almost 17,000 records. The features of the products are listed in Table 1, the features of the patient are listed in Table 2, while Table 3 is the Training accuracy of models after BPS.

**Table 1:** Number of features in the product.

**S/No**.	**Product features**	**Feature type**
1	Product barcode	Numeric
2	Product calories	Numeric
3	Product protein	Numeric
4	Product Fat	Numeric
5	Product sodium	Numeric
6	Product carbohydrate	Numeric
7	Product fiber	Numeric
8	Product cholesterol	Numeric

**Table 2:** Features of patients.

**S/No**.	**Patients features**	**Feature type**
1	Patient number	Numeric
2	Patient age	Numeric
3	Patient gender	Categorical
4	Patient weight	Numeric
5	Patient disease	Categorical
6	Patient calories	Numeric
7	Patient protein	Numeric
8	Patient fat	Numeric
9	Patient sodium	Numeric
10	Patient carbohydrate	Numeric
11	Patient fiber	Numeric
12	Patient cholesterol	Numeric
13	Target class	Categorical

**Table 3:** Training accuracy of models after BPS.

**Model name**	**Average accuracy %**
MLP	92.91
RNN	94.5
GRU	95.29
LSTM	96.5
Naïve Bayes	92.81
Logistic Regression	92.79

### Data Preservation and Protection

**Figure 1:**
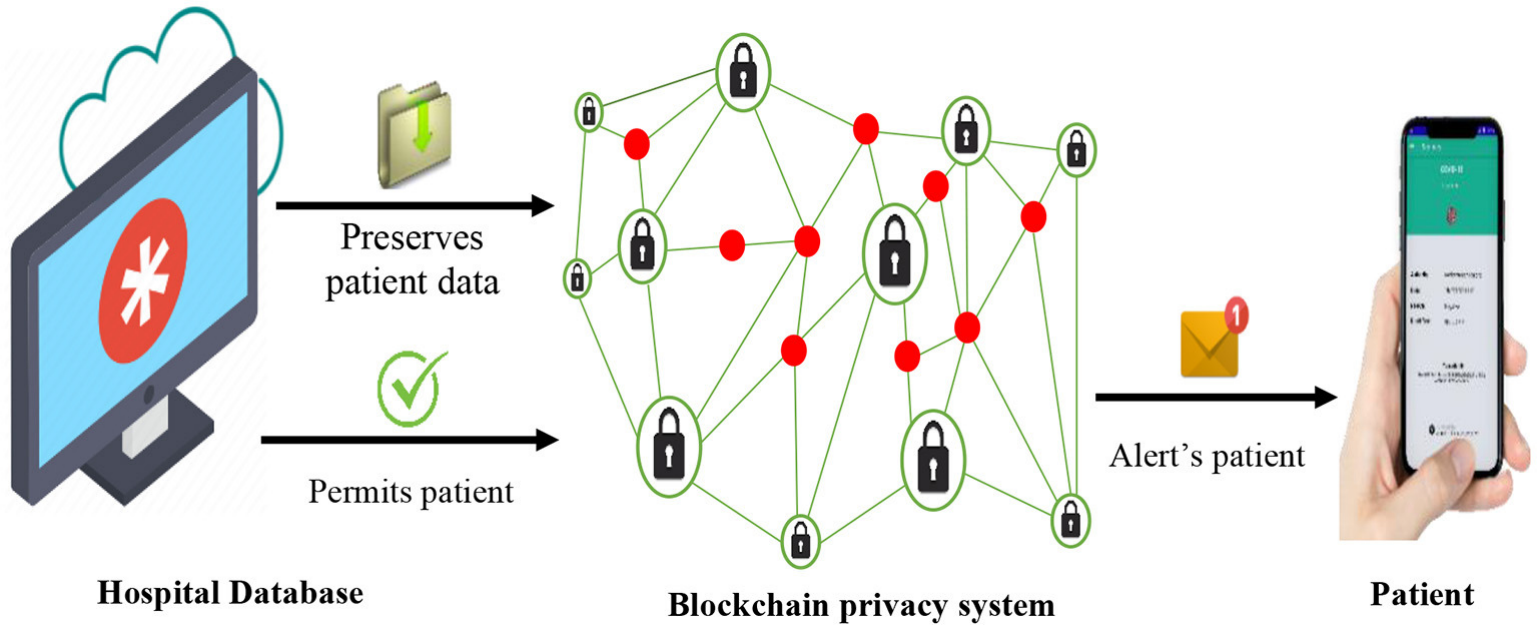
BPS-based data storage and patient alert preservation system.

The proposed BPS can be used by patients to store and preserve their personal data which are applicable to recommender systems. Example of such data includes diagnosed illness, treatment status, demographic attributes, prescribed medications, and response performance, among others. The BPS stores and fully encrypts this set of data, such that, accessing them without permission is almost impossible. Other related data, such as health history, diets of the patients, and more, which illustrates the actual treatment performance that is vital for the recommender systems are classically gathered by the hospital database system (HDS). These user-sensitive data are transmitted and stored in the BPS instead of the database of the hospitals, as illustrated in Figure 1, in order to enhance privacy by preventing attacks of the eavesdroppers, hacker attacks, and other intruding vulnerabilities. On the other hand, the BPS grants adequate permission to the patients to access these data in a very secured and trusted format. In case of a recommendation, prescription, check-ups, or emergencies, the BPS also sends a prompt notification to the registered smart device of the patient. This ensures adequate transparency about the data collection events of the hospitals for the patients (users).

### Cooperative Filtering

**Figure 2:**
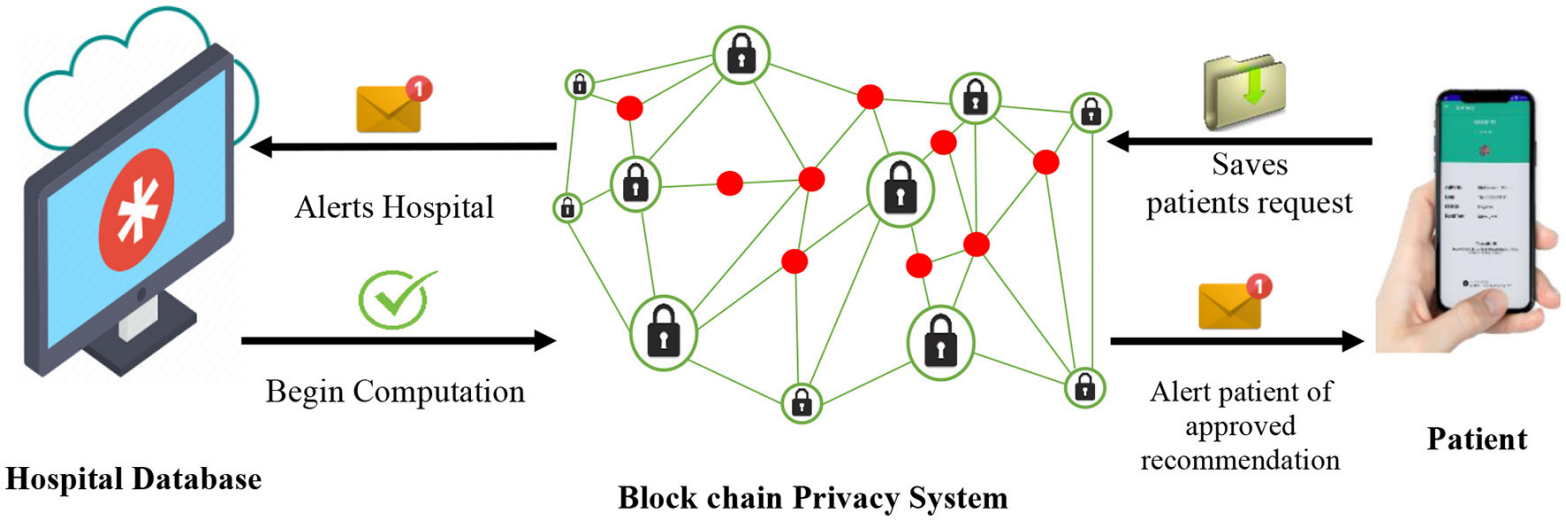
BPS-based data storage, hospital management and preservation system.

One of the major advantages of the BPS over other existing privacy technology is its ability to perform efficient data computations while keeping all input data confidential. As illustrated in Figure 2, the hospital is notified each time the patient (user) attempts to obtain a recommendation for their confidential medical details. Because the hospital runs a multi-user system with several of its patients, their individual data are employed as input variables for the cooperative filtering algorithm of the recommender systems to sort and locate the best recommendations for each enquiring patient. Meanwhile, the hospital database manager can only view basic information of the patient as the rest of the sensitive data and computations are performed at the BPS. In conclusion, the patient gets a notification alert and receives secure permission to access the subsequent recommendations.

### Data Processing

#### Data Normalization

Functions of data cleaning are carried out to get rid of noise and regularize the features after choosing a dataset. The aim of regularization is for the dataset to be scaled into a single range. This is done because the dataset has varied scale values. They can be one-digit, two-digit, or three-digit values so every value is brought into one scale so that the machine learning models perform more effectively. For this reason, we carried out min-max normalization. In this study, min-max scaling normalizes values within the scope of [0, 1] and is illustrated in Equation (1) below.


(1) $$\begin{array}{l} N_{i}=\frac{c_{i}-min\left(c_{i}\right)}{max\left(c_{i}\right)-min\left(c_{i}\right)} \end{array}$$

where *y* = *(C*_1_*, C*_2_… *C*_*n*_*)* are the number of features. *C*_*i*_ is the feature to be normalized and *N*_*i*_ stands for the features that have been formalized. As a result of doing this, all the features now have identical weights, and they are all in a single scope.

#### Data Encoding

While this research was carried out, values that were duplicated and inconsistent were eliminated from the dataset before data encoding was performed. Subsequently, the nominal features are transformed into arithmetic values. The objective of this is to ensure that the backend functions of the machine learning models are performed using arithmetic values prior to their implementation. In this study, non-arithmetic data was transformed to arithmetic data prior to the performance of data encoding. Machine learning (ML) algorithms backend computations were also carried out on arithmetic values before data was passed to the suggested model.

#### Optimal Feature Visualization

A Product calorie has an important percentage of 48% in the dataset. Product fat has a significance percentage of 12%. Product protein and carbohydrates have significance percentages of 8%. Product sodium has an important percentage of 6%. The user number has an important percentage of 5%. Furthermore, product fiber, user fat, and protein have significance percentages of 2% each. Age, user weight and calories, disease, product barcode, and user carbohydrate have important percentages of 1% each in the dataset. Random Forest is a combination of decision trees. For the prediction to be correct, it integrates all the decision trees and gives more precise results. It is used not just for regression and classification, but also in the application of the best features in the dataset. Its best attribute is that regression and classification can be performed using it. When classifying Random Forest, the majority vote was utilized in the prediction of the target. However, for the evaluation of regression, Random Forest takes the average value of all the decision trees and predicts it as a threshold as stipulated for each node. Subsequently, there is a performance of splitting based on that threshold. The threshold is stipulated by computing gain-index and entropy. Their various equations are shown below.


(2) $$Entropy:\;K(b_{1},b_{2},\ldots b_{s})=\sum_{i=1}^{s}(b_{1}\;log(\frac{1}{b_{1}})$$

where b_1_, b_2_…b_s_ stands for the probabilities of the class labels.


(3) $$\begin{array}{l} Gain\left(R,S\right)=K\left(R\right)\sum_{i=1}^{S}x\left(R_{1}\right)K\left(R_{1}\right) \end{array}$$

### Deep Learning Classifiers

#### Multilayer Perception

There exist various kinds of neural networks created and developed previously. All the existing ANNs may be distinguished by their processing unit (PE) transfer functions. Their techniques of learning and by the link equations. Processing unit is a basic constituent of ANN. It collects numerous signals that are weighted from other existing processing units. The framework of forwarding ANN is like this. The first layer has its input units. There are hidden layers in the middle. Finally, the last layer comprises output units. The function of the input unit is the provision of data from outside sources. Next, the data that is collected is transported to concealed layers where it is amplified with weights. Finally, it is passed to the output layer to initiate the final signals. The classification capacity of ANN is based completely on concealed layers. These concealed layers are linked by the synapses with the neighbor's layers. If there are *p* input data points (b_1_, b_2_… b_p_), we call it *p* number of features in the data. In the ANN framework, all the features are amplified with weights (e_1_, e_2_…e_p_) and then added as is stated in Equation (4) below.


(4) $$\begin{array}{l} E.B\;=\;e_{1}b_{1}+\;e_{2}b_{2}+...+e_{p}b_{p}\;=\sum_{1=1}^{p}e_{i}b_{i} \end{array}$$

where “*p”* represents the total amount of features present in the dataset given as input B to the input layer. While “*e”* stands for the weights of all the features amplified with its weight. It is referred to as the dot product too. The addition of the bias function is done next in the dot product function. It will lead to Equation (5) stated below.


(5) $$\begin{array}{l} a=\sum_{1=1}^{p}e_{i}b_{i}+bias \end{array}$$

In Equation (5), *a* denotes the activation function f (a). In this manner, we will obtain the output for the first neuron and the first hidden layer. This procedure will be repeated until the last weight and the last input is seen.

### Recurrent Neural Network

Recurrent Neural Network is a kind of ANN where node-to-node links create a graph administered along with a corporeal order which makes provision for the display of dynamic behavior. It is a type of new ANN that has memory cycles that are directed.

#### Long Short-Term Memory

This is a framework that carries out a memory augmentation for the RNN. In this study, 3 layers of LSTM with a batch size of 32, as well as the sigmoid function is used to activate it. The Adam function is utilized for optimization. Binary cross-entropy is used to compute function loss.

#### Gated Recurrent Units

The principle of the GRU is more current than the LSTM. Basically, it functions more effectively. It is quicker in the training of models as opposed to the LSTM. The model can be modified and manipulated easily. However, LSTM performs better than the GRU if a longer memory is needed. Eventually, the comparison of performance depends on the kind of dataset being used. The frameworks LSTM and GRU are somewhat similar, but there are some important differences stated below:

- The GRU comprises two gates, but the LSTM has three.
- Gated recurrent units do not have internal memory that is opposed to the detectable hidden form. The output gate which is embedded in LSTMs is also not present in the GRUs.
- There is no application of second non-linearity when calculating GRU output as opposed to the LSTMs.

### Machine Learning Classifiers

#### Logistic Regression

This is a popular classification algorithm utilized in machine learning. Basically, a bipartite result is acquired with it. Its purpose is to find a link connecting the possibility of a particular outcome and its features. A logit (odds) function is utilized in this algorithm. In Equation (6), the algorithm is described as seen below:


(6) $$\begin{array}{l} \log\left(\frac{r\left(b\right)}{1-r\left(b\right)}\right)=\;\beta_{o}+J_{1b} \end{array}$$

In the equation above, the logit function is $\log\left(\frac{r\left(b\right)}{1-r\left(b\right)}\right)$ and the odd function is $\left(\frac{\text{r}\left(\text{b}\right)}{1-\text{r}\left(\text{b}\right)}\right)$. The odds show the probability proportion of insertion of the feature to the likelihood of failure or lack of features. Output is normally done in this algorithm subsequent to the mapping of inputs to log-odds in a straight blend. Now, the contrary of the function stated above would be;


(7) $$\begin{array}{l} R\left(B\right)=\;\frac{l^{\beta_{o}+\;\beta_{1b}}}{1+\;l^{\beta_{o}+\beta_{1b}}} \end{array}$$

Equation (7) is referred to as a sigmoid function which is a mathematical function that has an “S”—shaped curve characteristic, which converts the values between the range 0 and 1. As long as the probability value is initiated within the range of 0 < *R* < 1, then we chose the variables in the logarithm in a manner to expand the likelihood of scrutinizing sample values.

#### Naive Bayes

This is a collation of algorithms that have a joint where all the pairs of the features are unconstrained. Two hypotheses are considered in the algorithm which are as follows:

1. All the functions are separate.
2. The texts need to be translated into arithmetic values in the instance of features in text format. Bayes' Theorem is shown in Equation (8) below:

(8) $$\begin{array}{l} R\frac{G}{H}=\;\frac{R\left(\frac{G}{H}\right)R\left(G\right)}{R\left(H\right)} \end{array}$$

where G and H events, R (G) is the prior probability $R\left(\frac{G}{H}\right)=H^{\prime }s$ posterior probability. Now, Bayes' theorem can be implemented by Equation (9) as seen below:


(9) $$\begin{array}{l} R\frac{v}{B}=\;\frac{R\left(\frac{B}{v}\right)R\left(v\right)}{R\left(B\right)} \end{array}$$

where B represents the dependent feature vector and v is the class variable, B is of size n so, B = (B_1_, B_2_, B_3_ …, Bn). For evidence to be split into unconstrained categories for events G and H: R (G, H) = R (G) R (H). The results then become:


(10) $$\begin{array}{l} R\left(\frac{v}{B_{1}\ldots n}\right)=\;\frac{R\left(\frac{B_{1}}{v}\right)R\;\left(\frac{b_{2}}{v}\right)\ldots \ldots R\left(\frac{b_{n}}{v}\right)}{R\left(B_{1}\right)R\left(B_{2}\right)\ldots \ldots R\left(B_{n}\right)} \end{array}$$

It can be expressed as Equation (11) below:


(11) $$\begin{array}{l} R\left(\frac{v}{b_{1}\ldots n}\right)=\;\frac{R\left(v\right)\Pi_{i=1\;}^{n}R\left(\frac{B_{i}}{v}\right)}{R\left(B_{1}\right)R\left(B_{2}\right)\ldots \ldots R\left(B_{n}\right)} \end{array}$$

Because the denominator is continuous for input, so in Equation (12) below:


(12) $$\begin{array}{l} R\left(\frac{v}{b_{1}\ldots n}\right)=\infty R\left(v\right)\Pi_{i=1}^{n}R\left(\frac{B_{i}}{v}\right) \end{array}$$

For a classifier model to be produced, the input probabilities for v must be determined and the output with the highest probability has to be taken. Hence, in Equation (13) below:


(13) $$\begin{array}{l} v=argmaxR\left(v\right)\Pi_{i=1}^{n}R\left(\frac{B_{i}}{v}\right) \end{array}$$

Finally, what is left for calculation is $R\left(\frac{b_{i}}{v}\right)$ which is the initial probability and R (v) which represents the contrast of the logit function.

### Evaluation Metrics

Various metrics are utilized in the evaluation of the performance of our suggested model. These are stated below:


(14) $$\begin{array}{l} Accuracy=\;\frac{A_{z}+A_{n}}{A_{z}+C_{z}+C_{n}+A_{n}} \end{array}$$

The objective of precision is to examine the True Positive (AZ) units in connection to False Positive (CZ) units.


(15) $$\begin{array}{l} Precision=\;\frac{A_{z}}{A_{z}+C_{z}} \end{array}$$

The objective of recall is to examine True Positive (AZ) units in connection to False Negative (CN) units that are not classified. The arithmetic arrangement of recall is stated in Equation (16) below:


(16) $$\begin{array}{l} Recall=\;\frac{A_{z}}{A_{z}+C_{n}} \end{array}$$

Sometimes, assessment of performance may not be very efficient with recall and accuracy. For example, if a mining algorithm has high precision but low recall, then another algorithm is needed. Then comes the question of which algorithm is more effective. This challenge is solved utilizing F1-measure that gives a mean recall and precision. F1-measure can be calculated as follows:


(17) $$\begin{array}{l} Measure=\;\frac{2\;\times Precision\times Recall}{Precision+Recall} \end{array}$$

### Software Analysis

A Colab software installed on a Core-i7 computer system is employed in performing the Deep Learning experiments. The system is fully equipped with 16GB RAM, 4 CPUs, a 1.7 GHz processor, and about 20GB obtained from Google Colab laboratory. The overall dataset was partitioned into three segments which includes training segment, cross-validation segment, and testing segments. Constituting 80% of the dataset was utilized in the research as the training set, 20% was used as the testing set, while *K*-Fold Cross-validation was employed and is applicable to both the training and testing sets.

The study employed both machine and deep learning classifiers in this research and their training accuracies are presented in Table 4. Using a 32-batch sized 3-layer LSTM with sigmoid activation function, it is established that the LSTM classifier ranked maximum with 95.45% training accuracy, while MLP ranked as the least at 86.5%. The training accuracies for the rest classifiers which are GRU, RNN, Logistic regression [LR], and Naive Bayes were at 94.6, 92.3, 88.5, and 87.2% training accuracies, respectively.

**Table 4:** Performance of training score accuracy for selected classifiers.

**Classifier**	**Percentage accuracy (%)**
LSTM	95.5
RNN	92.3
MLP	86.5
Logistic Regression	88.5
Naive Bayes	87.2
GRU	94.6

## Experiments and Results

**Figure 3:**
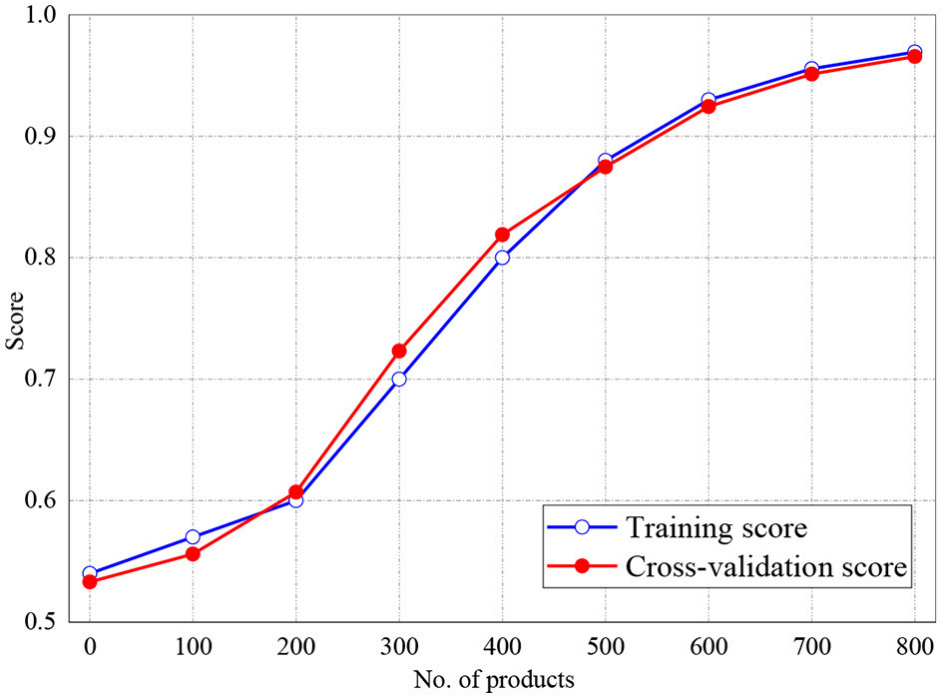
Result for Training and Cross-Validation of Naive Bayes classifier.

**Figure 4:**
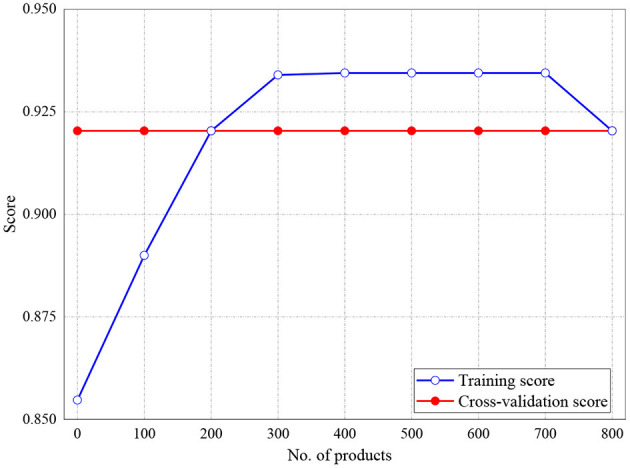
Result for Training and Cross-Validation Scores of Logistic Regression classifier.

**Figure 5:**
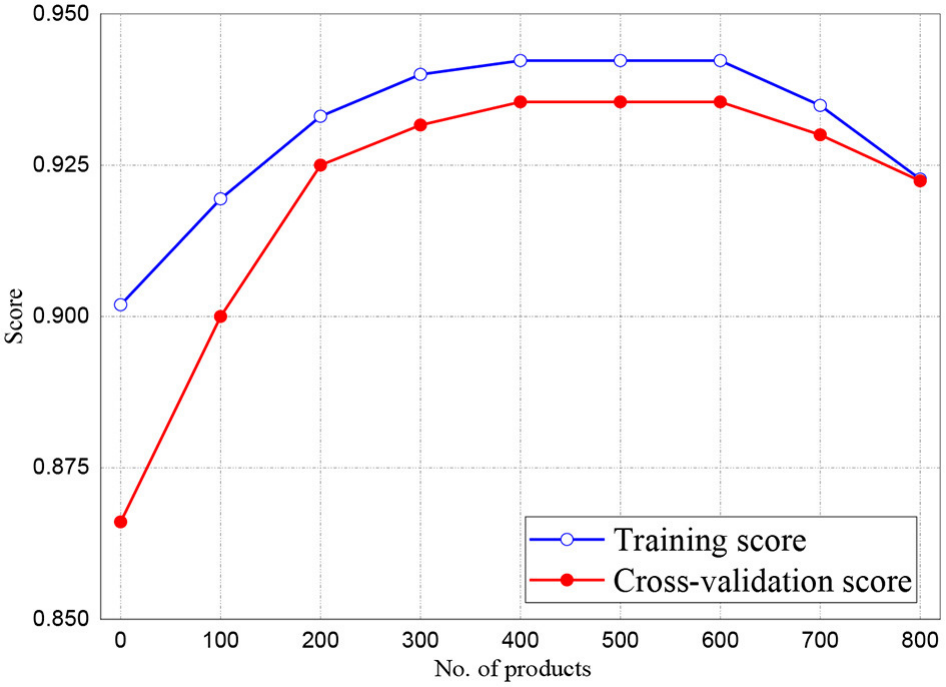
Result for Training and Cross-Validation of MLP classifier.

The training and validation scores of Naive Bayes, LR, and MLP classifiers are presented in Figures 2, **4**, **5**, respectively. For all the figures, the training scores are denoted with a blue line/curve while the red curve represents the scores of cross-validations. For Naive Bayes in Figure 3, it is observed that the training and cross-validation scores merged at 87.2%. The cross-validation score of the logistic regression in Figure 4 remained stable and linear while the training score increases up to 93.8%, stabilizes, and further decreases. The validation and training scores of the MLP classifier is illustrated in Figure 5. The experiment shows that both the training and validation scores of the MLP classifier training scores increase at some point and ultimately decreased.

The test accuracies of the MLP classifiers are presented in Table 5. The result shows that the LSTM classifier realized a maximum of 99.5% test accuracy, while the MLP classifier achieved the least score of 90.3% test accuracy. The rest classifiers which are GRU, Naive Bayes, RNN, and LR all attain 98.8, 95.6, 95.5, and 90.8% testing accuracies, respectively.

**Table 5:** Performance of testing score accuracy for selected classifiers.

**Classifier**	**Percentage accuracy (%)**
LSTM	99.5
RNN	95.5
MLP	90.3
Logistic Regression	90.8
Naive Bayes	95.6
GRU	98.8

**Figure 6:**
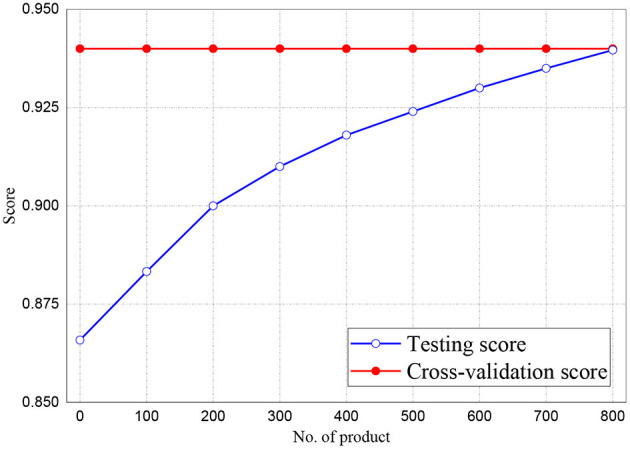
Result for Testing and Cross-Validation of Naive Bayes classifier.

**Figure 7:**
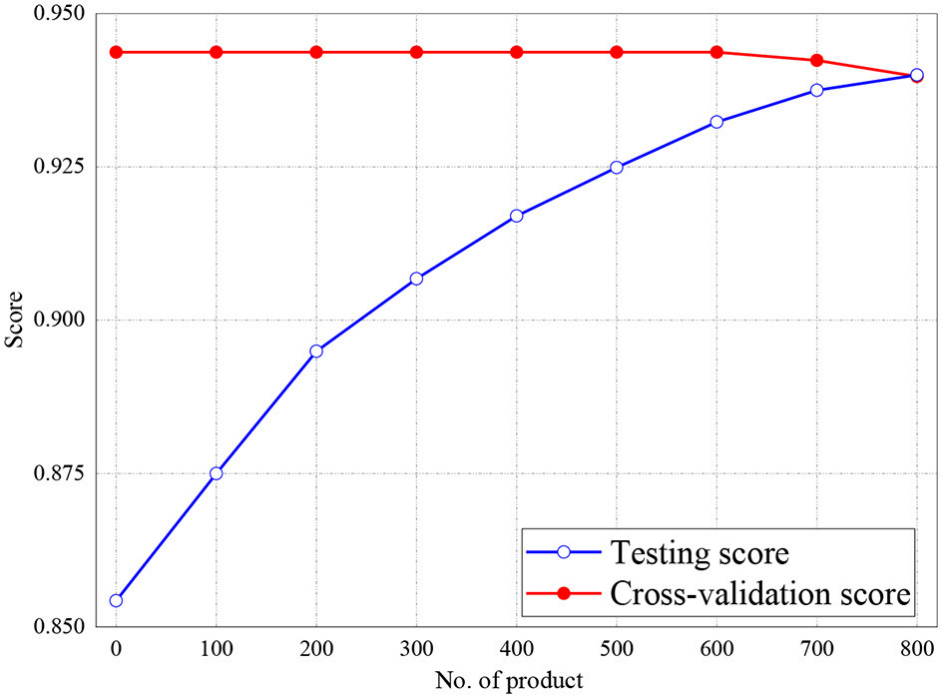
Result for Testing and Cross-Validation of Logistic Regression.

**Figure 8:**
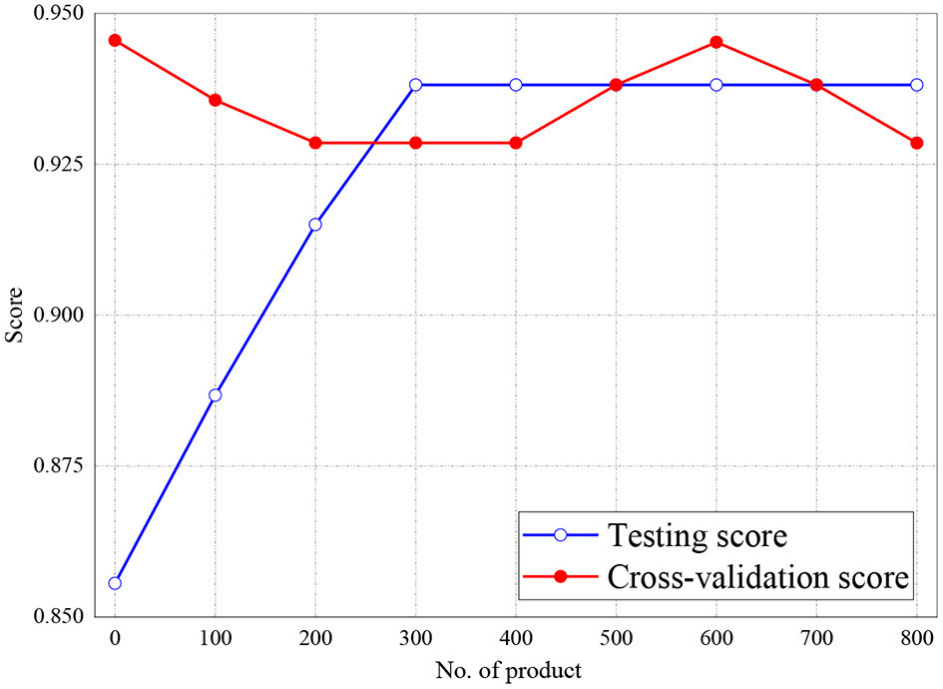
Result of Testing and Cross-Validation for MLP classifier.

Figures 6, 7 represent the testing accuracies and cross-validation scores of both Naive Bayes and LR models, respectively, it is observed that the testing accuracies and validation scores for both models merged at 94 and 94.28%, respectively. Similarly, Figure 8 shows that the validation and testing scores for MLP merged at 93.81 and 92.85%, respectively.

**Figure 9:**
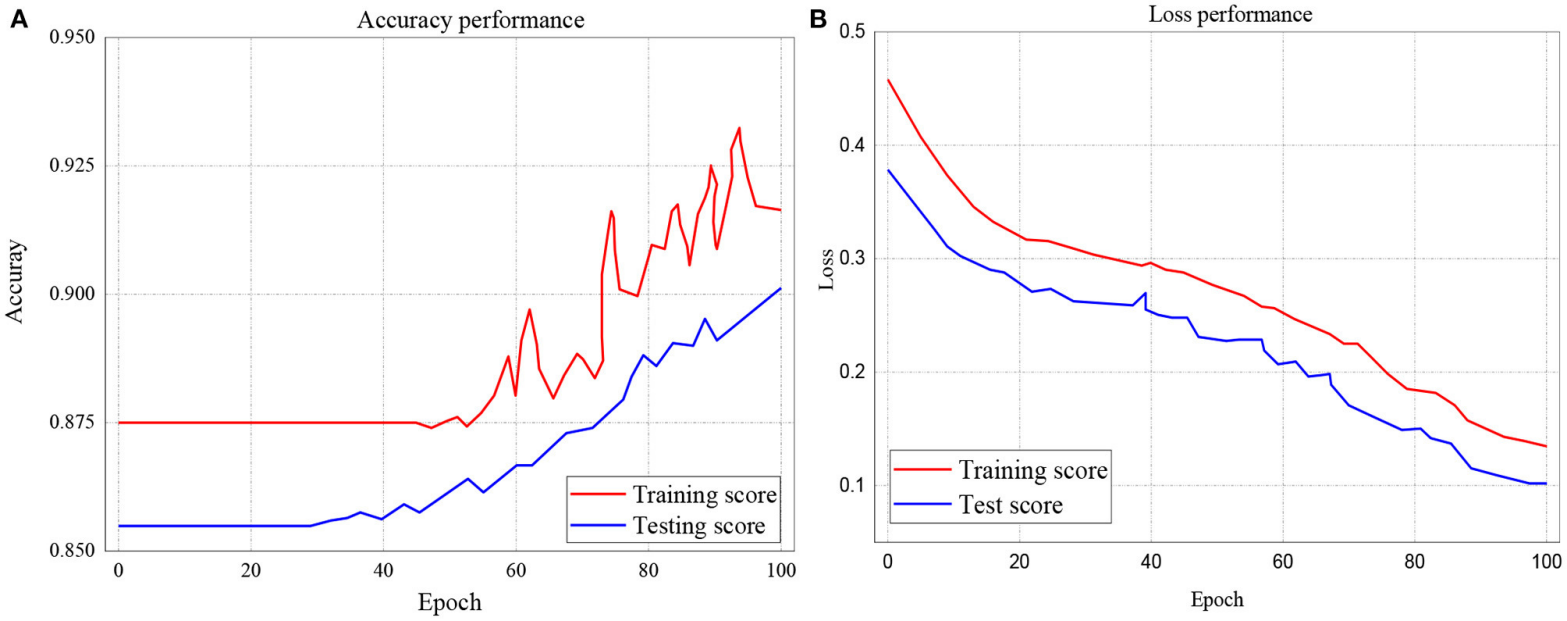
**(A,B)** Performance accuracy and loss of Training and Testing Scores for GRU classifier.

**Figure 10:**
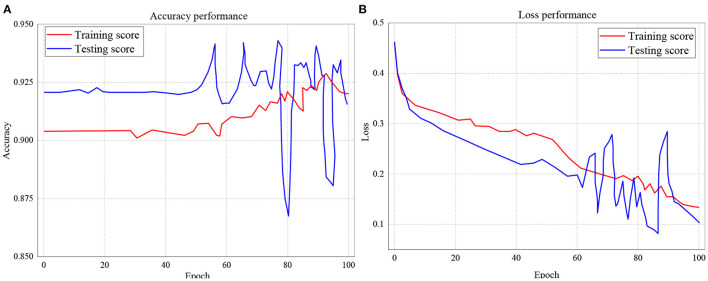
**(A,B)** Performance accuracy and loss of Training and Testing Scores for LSTM.

**Figure 11:**
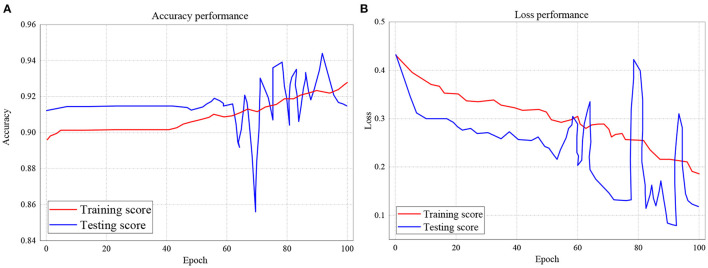
**(A,B)** Performance accuracy and loss of Training and Testing Scores for RNN.

The study further investigated the accuracies and loss performance of training scores against the testing scores for all the models. The results of these investigations are presented in Figures 9–**11**. The red curves denote the training scores, while the blue curves denote the testing scores for both the accuracy and loss performances. Figure 9A represents the performance accuracy of the training and testing scores for the GRU classifier. It is observed that the performance started from 87.5% and remained stable until after about 45 epochs. It further witnessed instabilities till it achieved an apex of 93% at 96.6 epoch. Similarly, the blue curve which represents the testing score started and remains stable at 85.5%, witnessed several instabilities but attained its apex of 90.2% at 100 epochs.

On the other hand, the loss performance of the training and testing scores for the GRU classifier is represented in Figure 9B. The figure indicates that the loss performance of the training score began from 0.45 and decreased to 0.14. In the same way, testing loss performance commenced from 0.38 down to 0.102.

The training and testing scores accuracy for LSTM is presented in Figure 10A, while the loss performance for the same model is illustrated in Figure 10B. Considering Figure 10A, the experiment shows that for accuracy, the training score started from 92.1%, with the least performance of 86.8% and peak performance of 94.3%. Likewise, for testing score accuracy, the performance began from 90.4% to a peak of 92.8% with the least performance of 90.1%. On the other hand, the training and testing loss performance of the LSTM classifier is illustrated in Figure 10B. The performance indicates that the training loss score commenced from 0.46 and declines to 0.14 after 100 epochs. The testing loss performance shows that the reading also started from 0.46 but decreased down to 0.1 after 100 epochs.

In Figure 11A, the testing and training scores accuracy for the RNN model is presented, while the loss performance for the same model is illustrated in Figure 11B. With respect to Figure 11A, the experiment shows that for accuracy, the training score started from 89.5% and also records as the least performance with a peak performance of 92.9% after 100 epochs. Also, for testing score accuracy, the performance began from 91.3% to a peak of 91.5% with the least performance of 85.8%. On the other hand, the training and testing loss performance of the RNN classifier is illustrated in Figure 11B. The performance indicates that the training loss score commenced from 0.435 and declines to 0.185 after 100 epochs. The testing loss performance shows that the reading also started from 0.435 but decreased down to 0.12 after 100 epochs.

**Figure 12:**
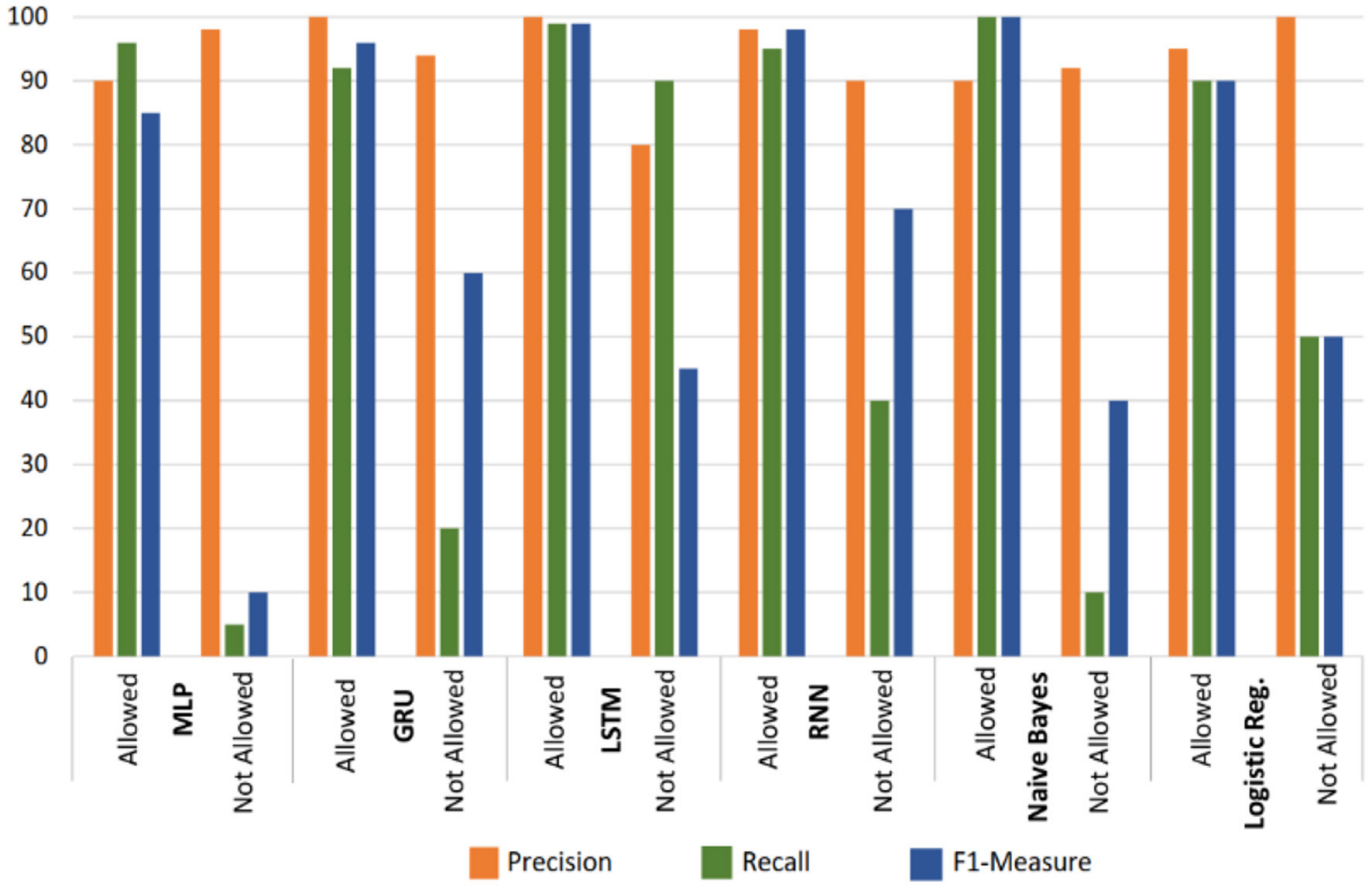
Result of the Classification for Deep and Machine Learning classifiers.

The classification further recorded the deep learning and machine learning classifiers which are presented in Figure 12. The result indicates that the LSTM classifier outperforms the rest of the tested classifiers with reference to recall, F1-measure, and precision. The experiment is divided into allowed and not allowed classes. The performance of the LSTM classifier for the allowed class is measured at 99, 100, and 99% for recall, precision and F1-measure accordingly. Meanwhile, the disallowed class performance of the LSTM classifier measure at 90, 80, and 45%, for recall, precision, and F1-measure, respectively. The rest of the experimented models performs well in terms of the allowed class. However, they did not perform well with the disallowed class.

## Conclusion and Future Work

The analysis of collected automated medical data has been established to have the potential in providing adequate recommendations for the improvement and management of patients with special needs in the hospitals through different algorithm developments and knowledge discovery. However, several medical facilities and special care patients are yet to grasp the usage and intent of secured recommender systems. One of the reasons for not embracing the systems is due to fear of data Privacy Breach. This research proposes a secure deep learning-based recommender system that estimates and issues basic treatment and diet recommendations to patients with special needs without revealing their sensitive health details. The system automatically issues exact recommendations to ailing patients based on their basic demography, diet, health history and other related data. The deep learning classifiers considered in this research include LSTM, MLP, GRU, RNN, Naive Bayes, and LR. The results of the performed experiments show that LSTM and GRU outperform every other classifier with reference to their recall, precision, and *F-*1 measures for both the allowed and not allowed classes. The performance of the LSTM classifier for the allowed class is measured at 100, 99, and 99% for precision, F1-measure, and recall, respectively. Meanwhile, the disallowed class performance of the LSTM classifier measures 90, 80, and 45% for recall, precision, and F1-measure, respectively. In the future, we shall incorporate the concept of multidimensionality in the secured diet recommender systems, where patients with special needs will maintain privacy after the whole process.

## Data Availability Statement

The raw data supporting the conclusions of this article will be made available by the authors, without undue reservation.

## Author Contributions

All authors listed have made a substantial, direct and intellectual contribution to the work, and approved it for publication.

## Funding

This paper was supported by the key research and development plan (social development) projects BE2016630 and BE2017628 of Jiangsu province, the scientific research project Z201603 of Wuxi health and family planning commission.

## Conflict of Interest

The authors declare that the research was conducted in the absence of any commercial or financial relationships that could be construed as a potential conflict of interest.

## Publisher's Note

All claims expressed in this article are solely those of the authors and do not necessarily represent those of their affiliated organizations, or those of the publisher, the editors and the reviewers. Any product that may be evaluated in this article, or claim that may be made by its manufacturer, is not guaranteed or endorsed by the publisher.
